# Human-algorithm teaming in face recognition: How algorithm outcomes cognitively bias human decision-making

**DOI:** 10.1371/journal.pone.0237855

**Published:** 2020-08-21

**Authors:** John J. Howard, Laura R. Rabbitt, Yevgeniy B. Sirotin

**Affiliations:** Maryland Test Facility (MdTF), Upper Marlboro, Maryland, United States of America; Universitá Cattolica del Sacro Cuore, ITALY

## Abstract

In face recognition applications, humans often team with algorithms, reviewing algorithm results to make an identity decision. However, few studies have explicitly measured how algorithms influence human face matching performance. One study that did examine this interaction found a concerning deterioration of human accuracy in the presence of algorithm errors. We conducted an experiment to examine how prior face identity decisions influence subsequent human judgements about face similarity. 376 volunteers were asked to rate the similarity of face pairs along a scale. Volunteers performing the task were told that they were reviewing identity decisions made by different sources, either a computer or human, or were told to make their own judgement without prior information. Replicating past results, we found that prior identity decisions, presented as labels, influenced volunteers’ own identity judgements. We extend these results as follows. First, we show that the influence of identity decision labels was independent of indicated decision source (human or computer) despite volunteers’ greater distrust of human identification ability. Second, applying a signal detection theory framework, we show that prior identity decision labels did not reduce volunteers’ attention to the face pair. Discrimination performance was the same with and without the labels. Instead, prior identity decision labels altered volunteers’ internal criterion used to judge a face pair as “matching” or “non-matching”. This shifted volunteers’ face pair similarity judgements by a full step along the response scale. Our work shows how human face matching is affected by prior identity decision labels and we discuss how this may limit the total accuracy of human-algorithm teams performing face matching tasks.

## Introduction

Government and business applications that must establish the identity of individuals frequently rely on photo identification documents issued by official government agencies. Authorized agents review these identity documents to ensure that the photo on the document matches the individual who presented it. This process of verifying a person based on their photo relies on a human cognitive task known as face matching. Humans perform face matching tasks hundreds of times every day and, like all human tasking, this activity is subject to errors and cognitive biases. For example, despite excellent ability to recognize familiar faces, people are generally poor at face matching tasks involving unfamiliar faces, with error rates in excess of 10% [1–4].

Recent advances in machine learning have led to the development of computer-based face matching algorithms capable of identifying individuals with accuracy rates rivaling even those of trained forensic facial examiners [3, 5]. Nonetheless, humans continue to be a necessary part of most identity tasks, particularly when the outcome of the task is impactful. The reasons for continuing to have this “human-in-the-loop” are two-fold. First, humans are uniquely capable of deciding when the use of automated face matching is not appropriate. Second, humans are necessary to perform any exception processing steps, should the computer based technology fail. The level of effort associated with human review of automated face matching tasks can also vary. For example, forensic facial examiners may have weeks or months to review face images while security personnel may be required to decide if a face pair is a match in a few seconds or minutes.

Despite the rapid proliferation of face recognition technology in “human-in-the-loop” applications, the effect of incorporating algorithm decisions into human face matching tasks remains poorly understood. Questions outstanding include how human face matching is affected by information provided by algorithms and the conditions required for synergistic human-algorithm teaming.

### Prior work

Humans posses dedicated neural resources to process and recognize faces [6], with specific brain pathways for establishing face identity driven by differential neural activation. The sophisticated architecture dedicated to processing and remembering faces shows the social importance and evolutionary necessity of inferring information from faces. However, human performance on these tasks is known to be affected by long-term perceptual learning. For example, humans are generally poor at matching unfamiliar faces [7], as seen in the so-called “other race” effect, whereby an individual’s face discrimination performance is reduced when evaluating faces from people belonging to a racial category for which the individual has less prior experience [8]. Human face matching accuracy can also be impacted by short-term face adaption effects whereby the perception of a face can be altered by previously viewed faces [9–11].

Ancillary information can also influence human face matching judgements under uncertainty. Dowsett and colleagues documented beneficial effects of group decision-making for individuals working in pairs on face matching tasks, however, pair performance was limited by the performance of the best performing individual or fell below expectations if each individual’s errors were independent [12]. Fysh and Bindemann showed that identity labels (same, different, or unresolved) provided together with face pairs modulated face matching accuracy [13]. The authors theorized that labels may have drawn attention away from the face stimuli, increasing accuracy when label information was correct, but decreasing it when label information was incorrect. Indeed, spatial attention is known to improve human face discrimination accuracy, suggesting that adding uninformative labels should reduce face discrimination accuracy by drawing attention away from the face [14].

Finally, humans can confuse faces of people matched in gender, age and race. This explains why the majority of face pairs used on the GFMT are demographically homogeneous [2]. Additionally, in a White et al. study, participants were tasked with matching a probe face to a candidate list of eight faces selected by an algorithm based on computed similarity to the probe. The results of this study showed that people perform poorly at this task, achieving less than 70% correct performance, with a significant number of errors due to within demographic group false positives [15].

Given that these effects can lead to decreased human performance at face matching tasks, its not surprising that some have postulated using computer based face recognition algorithms for some face matching tasks. Indeed modern face recognition algorithms have been shown to outperform humans in matching unfamiliar faces [3, 5]. However, automated face recognition is not infallible. Computer algorithms can confuse faces of people matched in gender, age, and race with greater likelihood [16]. Computer based face recognition can also be fooled by liveness or spoofing attacks, where a non-biological sample, such as a photograph, is presented. Particularly when the impact of a face recognition system failure is high, full automation may be unwise. In these situations there is still a need to include a “human-in-the-loop”, in such a way that the face recognition output is combined with the human decision. The human operator can also complete a number of tasks that machines cannot do yet (e.g., detect suspicious behaviors or rule-out a match based on other information) or implement alternative processes/technologies should a face recognition system encounter a known failure case (e.g. a mask). However, there is little research on how human decision making is influenced by face recognition algorithms. Do the human performance numbers as reported in [7, 14, 15] and others improve when algorithm outcomes are provided? Fysh and Bindemann [13] conducted preliminary research on this and showed identity labels indeed shifted human determinations but postulated this was because of decreased attention to the face matching task. We reproduce their results but implement a signal detection theory framework to show that they are not due to decreased attention but instead arise because the algorithm information introduces a cognitive bias that shifts the human’s perception of face similarity.

### Applying signal detection theory to face matching tasks

Human performance on perceptual tasks like face matching can be modeled using signal detection theory (SDT) [17]. SDT posits that task performance is determined by the magnitude of the response stimuli generated along some perceptual scale internal to the observer. In the case of face matching, the scale is related to the similarity between the two faces. Similar faces may generate a high response and dissimilar faces may generate a low response. According to SDT, the observer makes a decision regarding whether a given pair of faces belong to the same person or to different people by comparing the value of the generated response to some internal criterion, or decision threshold. A particular stimulus (i.e. face comparison) response value above the individual’s criterion results in a “same person” determination, while responses below this threshold result in a “different people” determination. While the real distributions of responses for same and different pairs are hard to estimate, SDT allows the estimation of several convenient measures relevant to task performance, including a measure of the observers’ sensitivity to the separation between the two distributions (*d*′) as well as the location of the decision threshold, a receiver operating characteristic (ROC) curve that measures how performance changes given different thresholds, and a net threshold-independent measure of sensitivity tabulated as the area under the ROC curve (AUC). The *d*′ and the decision threshold can be readily estimated by measuring the likelihood that observers correctly classify face pairs that are the same, and measuring the likelihood that observers incorrectly classify face pairs that are different as the same. Data from tasks which ask observers to rate the similarity of face pairs along a scale can be used to further calculate the ROC and AUC.

Changes in decision threshold cognitively biases observers’ decision-making. Observers with high thresholds are more likely to classify face pairs as different, regardless of the truth, whereas observers with low thresholds are more likely to classify face pairs as the same. For example, groups of individuals who have exceptional face recognition abilities, or super-recognizers (SRs), have a greater sensitivity to face similarity than the general population [3, 18]. However, SRs also have higher decision thresholds when performing challenging face matching tasks [19]. In addition to differences in decision thresholds across individuals, face matching decision thresholds for a single individual can also change based on task structure. In a mock line-up task, observers matching sequentially presented faces had higher decision thresholds than those matching faces presented simultaneously [20]. These studies demonstrate how SDT analyses can be applied to face matching tasks.

Ancillary information, such as identity labels, can separately affect face sensitivity (i.e. discriminability) and decision thresholds in face matching tasks. Ancillary information can divert attention away from the face pair, which should result in lower sensitivity for faces reflected in lower values of *d*′. On the other hand, ancillary information may change the decision threshold independent of sensitivity, cognitively biasing decision-making. Fysh and Bindemann computed sensitivity and decision thresholds for different stimulus conditions, but did not discuss their findings within an SDT framework [13]. In our study, we utilize this method as a means to characterize how prior identity information influences human face matching performance.

### Contributions

Our study characterizes the cognitive effects of providing explicit identity information from a human or a computer source on human face matching performance, making the following contributions to our understanding of human-algorithm teaming:

1. First, we replicate prior results [13] showing a strong influence of prior identity decisions on human identity judgements, with a sample of hundreds of diverse subjects.
2. Second, despite a greater reported trust in algorithm decisions reported by our test volunteers, we show that volunteers’ decisions are similarly affected by prior match/no-match labels whether they are described as coming from human or algorithm sources.
3. Third, we use a signal detection theory framework to show that this prior information does not reduce face discriminability as would be expected from reduced attention to the face pair and as suggested in [13]. Instead we show that prior identity information cognitively biases human decisions, shifting the response criteria by one full step along the similarity scale used.
4. Finally, we discuss how these effects likely reduce the potential synergies of human-algorithm teams in real-world scenarios.

## Materials and methods

To study the effect of prior identity information on human face matching performance we developed a face matching task modeled after the Glasgow Face Matching Task (GFMT) [2]. This task was administered to paid volunteers (“volunteers”) as part of a larger biometrics test. Limited details of this test are presented in this section. Full details are available elsewhere [21, 22]. All testing and data collection was approved by an Institutional Review Board.

### Test logistics

Data for this study was gathered over a five-day period in May 2019, with 40-50 volunteers tested in morning and afternoon sessions. During testing, volunteers first performed biometric transactions using a diverse set of biometric technologies as part of an unrelated experiment [23]. The face matching task was administered on paper to volunteers at the conclusion of each test session. Volunteers did not have a time-limit for completing the task, but most finished the full survey within 15 minutes. The data were acquired using the IRB protocol “Development and Evaluation of Enhanced Screening” number 120180237, approved by New England IRB.

### Face matching task

**Fig 1:**
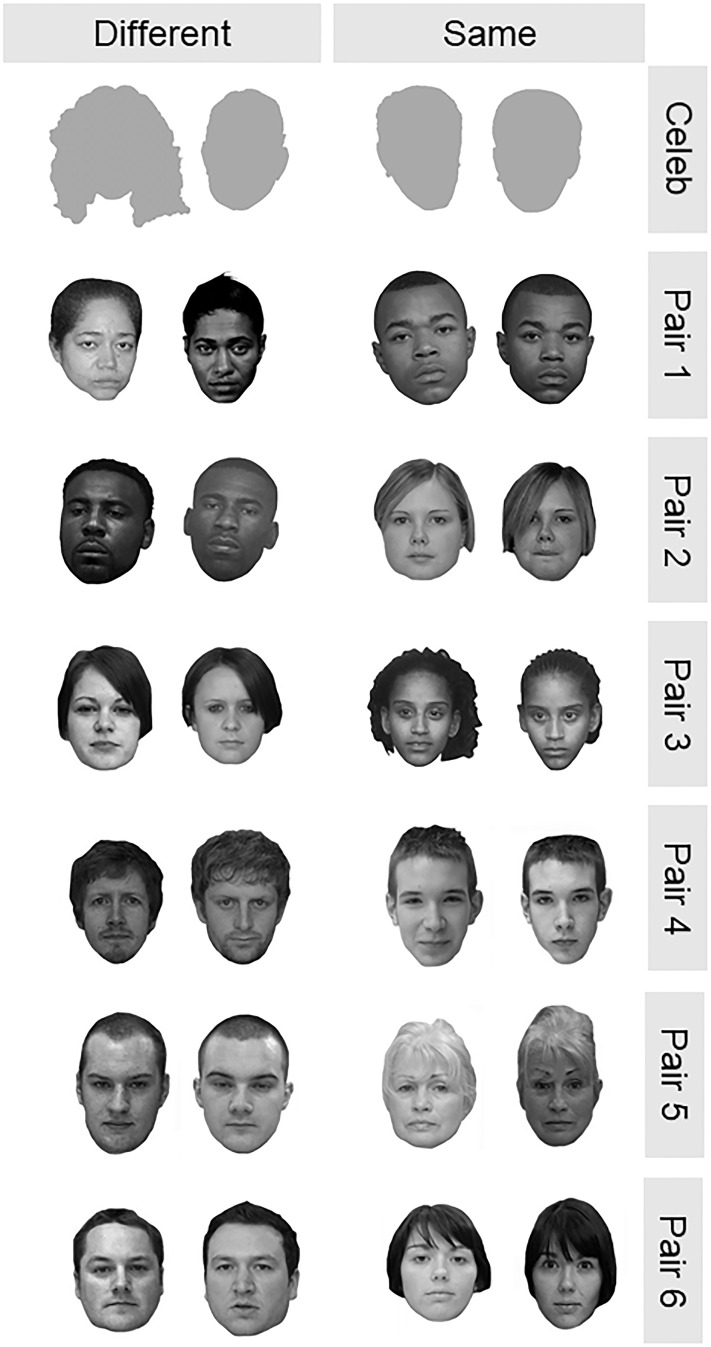
Image pairs selected for the face matching task. Celebrity faces not shown.

The face matching task administered as part of our study was modeled after the short version of the GFMT [2]. Our specific task was modified from the GFMT to include: prior identity information, diverse face pairs resembling our diverse test volunteer population, and easy to recognize celebrity faces. Due to time constraints, we were not able to administer the entirety of the GFMT-short version along with the additional face stimuli. We down-selected face pairs from the GFMT-short based on comparison scores generated by a commercial biometric face matching algorithm in order to select the most challenging same and different face pairs. To increase the diversity of face pairs in the task, we included African-American face pairs from the Multiple Encounters Dataset (MEDS) [24]. This was done as a means to account for the “other-race effect” [8]. Face pairs from MEDS were selected to have neutral expressions, and be as similar as possible based on similarity scores returned by a commercial face recognition algorithm. Selected faces from the MEDS data set were converted to gray scale and faces/hair were cropped to match the style of GFMT stimuli. Finally, as a screening control, we included a mated and an obviously non-mated doublet of celebrity face pairs. These face pairs are of well known, U.S. public figures, likely to be familiar to our test population recruited from the local area, and therefore easy to distinguish and match [7]. The full set of face pairs used as a part of our survey was balanced for the number of same and different faces as well as for gender. This set is shown in Fig 1.

The face-matching task was administered via paper with each face pair and decision presented on a separate page with a colored background and a 7-point certainty scale to indicate their match decision (Table 1). Faces in some pairs were from the same individual and in other pairs were from different individuals (Fig 1). All volunteers were told that their task was to review the face pair and provide their own judgment as to whether each face pair presented was of the same person or of different people. Volunteers could spend an unlimited amount of time reviewing each face pair and decision however, most volunteers completed the entire task within 15-minutes.

**Table 1:** Face matching task certainty scale.

Value	Response
3	I am absolutely certain this is the same person
2	I am mostly certain this is the same person
1	I am somewhat certain this the same person
0	I am not sure
-1	I am somewhat certain these are different people
-2	I am mostly certain these are different people
-3	I am absolutely certain these are different people

**Fig 2:**
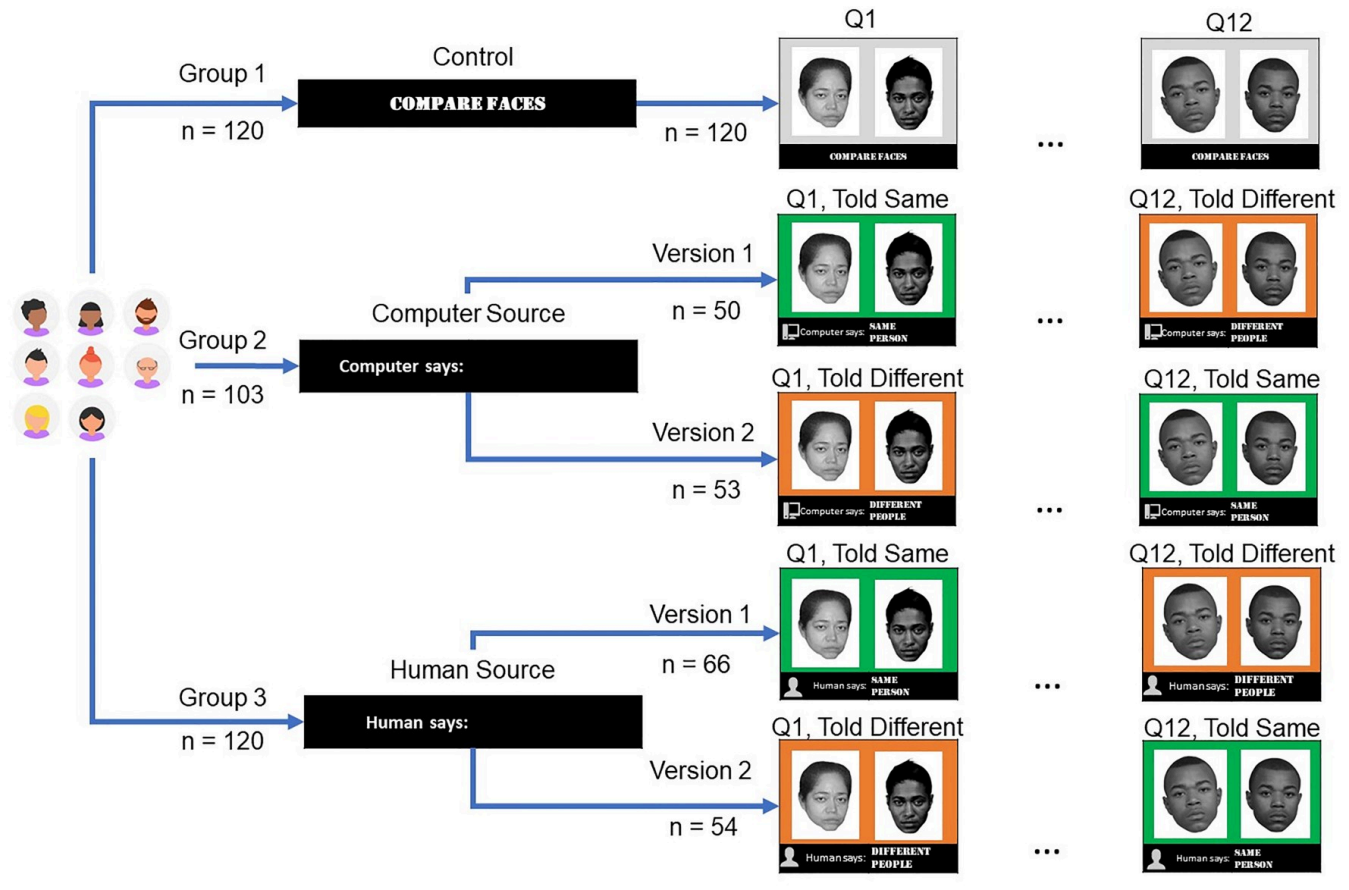
Survey experiment design. Images adapted from paper surveys.

The survey experiment followed a nested design (Fig 2). Volunteers were divided into three groups. Each group received a different survey variant (Control, Computer Source, Human Source). All survey variants presented volunteers with the same set of experimental face pairs (Q1 to Q12), in the same order, but with varying prior identity information context. In the control variant, no prior identity information was provided. In the Human Source variant, volunteers were told a human had reviewed each face pair and provided an identity decision. In the Computer Source variant, volunteers were told a computer had reviewed each face pair and provided an identity decision. In the Control survey variant, each face pair was presented on a grey background with the instruction “compare faces”. Prior identity information was nested within Computer Source and Human Source variants and indicated per face pair using a colored background and a text label (Told Same: green, “same person”; Told Different: orange, “different people”). Prior identity information was presented simultaneously to the volunteers with each face pair. Two versions (Version 1 and Version 2) of each survey with prior identity information ensured that each face pair was presented under both same and different instructions to half of the volunteers in each group.

At the beginning of the survey, volunteers were asked a multiple choice question regarding their trust in the ability of humans, computers, or themselves to identify a person. Volunteers in the control survey variant were asked, “Do you trust yourself to identify a person?”. Volunteers in the computer source variant were asked, “Do you trust a computer to identify a person?”. Volunteers in the human source variant were asked, “Do you trust a human to identify a person?”. Volunteers were given “Yes”, “No”, and “Not Sure” response choices. This question was used to assess trust in each information source.

### Volunteer demographics

A total of 376 paid volunteers participated in this study. Volunteers were recruited from the local area (Maryland, USA) as part of a larger evaluation of biometric systems [23]. Volunteers self reported gender, age, and race during pre-study screening and volunteer selection subsequently blocked on these categories to ensure equal representation across survey variants. Overall sample size was selected to ensure at least 100 unique volunteers took each survey variant. A total of 33 volunteers who failed to correctly respond on celebrity face pairs were excluded from analysis as incorrect responses suggested significant issues in face perception, lack of task understanding, failure to comply with instructions, or lack of familiarity with the celebrity faces. A total of 12 were excluded from the human source survey group, 13 from computer source survey group, 8 from control survey group. Responses to celebrity face pairs were not analyzed further.

Table 2 shows the demographics of the volunteers that were assigned to each survey condition, after removing volunteers that did not correctly respond to the celebrity face pairs. An equal number of volunteers self-reported their gender as “Male” and as “Female” and most volunteers self-reported as “Black or African-American” (shortened to “Black” in the Table 2) followed in frequency by those self reporting as “White”. For brevity, all other self-reported races are collapsed into the “Other” category. Blocking efforts were made to distribute gender, age, and race equally across the survey conditions. Table 2 shows this was successful in assigning statistically equal (Pearson Chi-squared test for count data) numbers of individuals to each survey variant. One subject was removed from Table 2 because they did not self-report demographic information.

**Table 2:** Blocking based on self-reported gender, age, and race within each survey condition.

Demographic Group	Survey Condition			Equal Distribution Across Conditions (*p* < 0.05)
	Computer	Human	None	
**Gender**				
Female	50	62	63	TRUE
Male	53	57	57	*χ*^2^(2) = 0.41,
				*p* = 0.81
**Age Bin**				
18-25	3	8	12	
26-35	35	37	36	TRUE
36-45	13	28	25	*χ*^2^(10) = 13.1,
46-55	27	18	23	*p* = 0.22
56-65	19	19	15	
65+	6	9	9	
**Race**				
Black	50	47	61	TRUE
White	32	48	47	*χ*^2^(4) = 8.3,
Other	21	24	12	*p* = 0.083

### Data analysis

Volunteer responses to each face pair were grouped based on participant and survey variant (Control, Computer Source, and Human Source). Within the Computer Source and Human Source groups, responses were additionally grouped based on nested prior identity information (Told Same vs. Told Different). Celebrity face pairs were presented last.

Signal detection theory metrics including receiver operating characteristic (ROC) curves and area under the curve (AUC) were calculated according to standard methods for rating tasks [25]. Briefly, we converted the graded certainty responses for each test item *R*_*i*_ to binary decisions (1 = same, 0 = different) using a sliding threshold *θ* ∈ (−2.5, −1.5, −0.5, 0.5, 1.5, 2.5), described in Table 3. For each threshold value, we calculated the true positive rate *TPR*_*θ*_, the false positive rate *FPR*_*θ*_, and overall accuracy *ACC*_*θ*_ as:

$$\begin{array}{c} \begin{array}{cc} & TPR_{\theta }=\frac{1}{n}\sum_{same}^{}R_{i}>\theta \\ & FPR_{\theta }=\frac{1}{m}\sum_{different}^{}R_{i}>\theta \\ & ACC_{\theta }=\frac{n\left(TPR_{\theta }\right)+m\left(1-FPR_{\theta }\right)}{n+m} \end{array} \end{array}$$ (1)

**Table 3:** Thresholds used for signal detection theory analysis.

*θ*	Description
2.5	very strict
1.5	mostly strict
0.5	slightly strict
-0.5	slightly permissive
-1.5	mostly permissive
-2.5	very permissive

The AUC was estimated as *Az* based on Gaussian signal and noise distributions assumptions [25] as:

$$\begin{array}{c} A_{z}=\Phi \left[\frac{I}{\sqrt{1+S^{2}}}\right] \end{array}$$ (2)

where *I* and *S* are the intercept and slope of the linear relationship between Φ^−1^[*HIT*_*θ*_] and Φ^−1^[*FA*_*θ*_], Φ^−1^ is the quantile function for the normal distribution, and Φ is the distribution function for the normal distribution. The two celebrity face pairs were not used in the calculation of SDT or accuracy metrics.

## Results

### Trust in identity information sources

**Fig 3:**
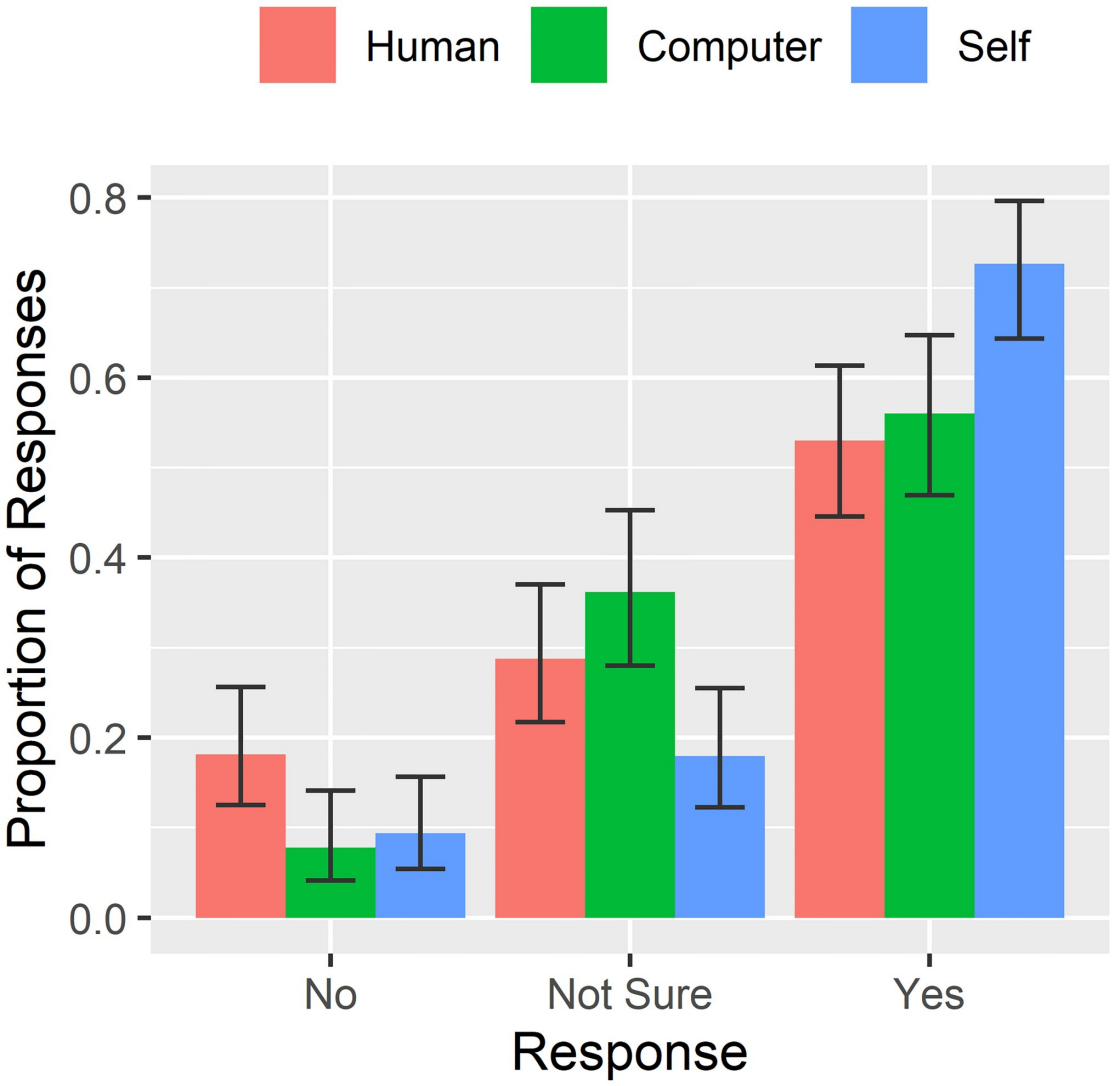
Perceived trust of identity sources. Error bars are binomial 95% confidence intervals.

To determine the degree to which individuals trusted each source of prior identity information (human versus computer), we tabulated the proportion of responses for each variant of the trust question (see **Face Matching Task**). These results are shown in Fig 3. We saw a significant difference in the proportion of “Yes” responses between different sources (*χ*^2^(2) = 12.0, *p* = 0.003). Overall, the responses indicated that most volunteers trusted themselves to make identity decisions (73% “Yes” responses), with lower but roughly equal proportions of volunteers trusting computers (56% “Yes” responses) and humans (i.e. other people; 53% “Yes” responses). Interestingly, there was also a significant difference in “No” responses between different sources (*χ*^2^(2) = 7.6, *p* = 0.022). Volunteers distrusted other people to make identity decisions at the highest rates (18% “No” responses) but similar proportions distrusted themselves (9% “No” responses) as computers (8% “No” responses). These data indicate that similar proportions of people trust identity information from computer and human sources, but a greater proportion of volunteers distrust identity information from other human sources.

### Variations in face matching accuracy based on prior identity information source

Using the response scale in Table 3, a decision threshold of *θ* = 0.5 separates responses indicating some confidence that an image pair is of the same identity from other responses. At this threshold, responses ranging from “absolutely certain these are different people” to “not sure” indicate a “different” determination by the volunteer and responses ranging from “somewhat certain this is the same person” to “absolutely certain this is the same person” indicate a “same” determination. The following sections use this single decision threshold to compare variations in matching accuracy across survey conditions. Later sections examine how performance varies across decision thresholds (see **Signal Detection Theory Analysis**). Differences in task performance associated with subject demographics are described in the accompanying S1 Appendix of this paper.

Overall, volunteers performing the face matching task in the absence of prior information were correct on 74% of face pairs (SD = 13, range 50%-100%), modestly lower than reported population norms for the short version of the GFMT (average 81% correct, SD = 10, range = 51%-100%, [2]). This difference in performance is reasonable given that our task included only “difficult” face pairs from the GFMT and MEDS datasets. This brings our average performance closer to what was reported in [15]. A one-way analysis of variance (ANOVA) of the accuracy numbers underlying the averages in Table 4 showed no effect of survey variant (none, human, or computer; *ACC*_0.5_; *F*(2) = 0.77, *p* = 0.46). This indicates that our paper-based 14-item task (12 evaluation pairs and 2 screening pairs) was consistent with the 40-item digital short version of the GFMT, and other previous work where algorithms select challenging face pairs.

**Table 4:** Face matching performance as a function of prior identity information source (threshold = 0.5). 95% confidence intervals are estimated by bootstrap resampling.

Source	n	ACC (95% CI)	FPR (95% CI)	TPR (95% CI)
None	120	0.75 (0.73–0.78)	0.19 (0.16–0.23)	0.70 (0.66–0.74)
Human	120	0.74 (0.72–0.77)	0.20 (0.17–0.23)	0.69 (0.65–0.73)
Computer	103	0.73 (0.71–0.76)	0.22 (0.18–0.26)	0.69 (0.65–0.72)

Table 4 also shows the false positive rate and true positive rate as a function of prior information source. A one-way ANOVA found no effect of survey variant on false positive rates (*FPR*_0.5_; *F*(2) = 0.55, *p* = 0.58) or true positive rates (*TPR*_0.5_; *F*(2) = 0.19, *p* = 0.83). This indicates that the *source* of identity information and the modifications in survey instructions did not influence the ability of volunteers to perform the face matching task at a threshold of *θ* = 0.5.

### Variations in face matching accuracy based on prior identity information

We next examined whether, as a population, volunteers changed their responses based on the prior identity information (i.e. what they were told about each face pair; responses to questions marked green versus orange in Fig 2) and prior identity information source (human vs. computer). For this analysis, we again used a threshold of *θ* = 0.5. Table 5 shows performance metrics as a function of prior identity information.

**Table 5:** Face matching performance as a function of prior identity decision (threshold = 0.5). 95% confidence intervals are estimated by bootstrap resampling.

Prior	n	ACC (95% CI)	FPR (95% CI)	TPR (95% CI)
None	120	0.75 (0.73–0.78)	0.19 (0.16–0.23)	0.70 (0.67–0.74)
Same	223	0.73 (0.71–0.76)	0.25 (0.22–0.29)	0.72 (0.68–0.75)
Different	223	0.75 (0.72–0.77)	0.17 (0.14–0.20)	0.66 (0.63–0.70)

The effect of prior identity information was nested within the human and computer survey variants and volunteers were presented same and different prior identity information for different face pairs on the same survey. The effect of prior identity information could not be examined for the control survey. We analyzed the data using a repeated measures ANOVA, examining the main effects of survey variant (between subjects) and prior identity information (within subjects). The ANOVA found no effect of prior identity information (same or different) on average accuracy for the experimental surveys (*ACC*_0.5_). However, it did find a significant effect of prior identity information on both true positive rates (*TPR*_0.5_; *F*(1, 222) = 6.56, *p* = 0.01) and false positive rates (*FPR*_0.5_; *F*(1, 222) = 14.76, *p* = 0.0002). True positive rates declined from 72% when volunteers were told face pairs were from the same person (green questions in Fig 2), to 66% when volunteers were told face pairs were from different people (orange questions in Fig 2). False positive rates declined from 25% when volunteers were told face pairs were from the same person, to 17% when volunteers were told they were from different people. These data are summarized in Table 5. This indicates that the prior identity decisions cognitively biased volunteers’ face matching decisions.

Interestingly, the ANOVA did not find any effects of survey variant (human or computer) on any of the measures. In fact, the average difference between *TPR*_0.5_ and *FPR*_0.5_ as a function of prior identity information was nearly identical. For the computer source, the difference in *TPR*_0.5_ was 0.058 and the difference in *FPR*_0.5_ was 0.087, nearly identical to 0.052 and 0.083, respectively, for the human source. This indicates that despite the fact that more volunteers reported greater mistrust of humans relative to algorithms to make identity decisions, volunteers were equally swayed by prior identity information from either source.

**Fig 4:**
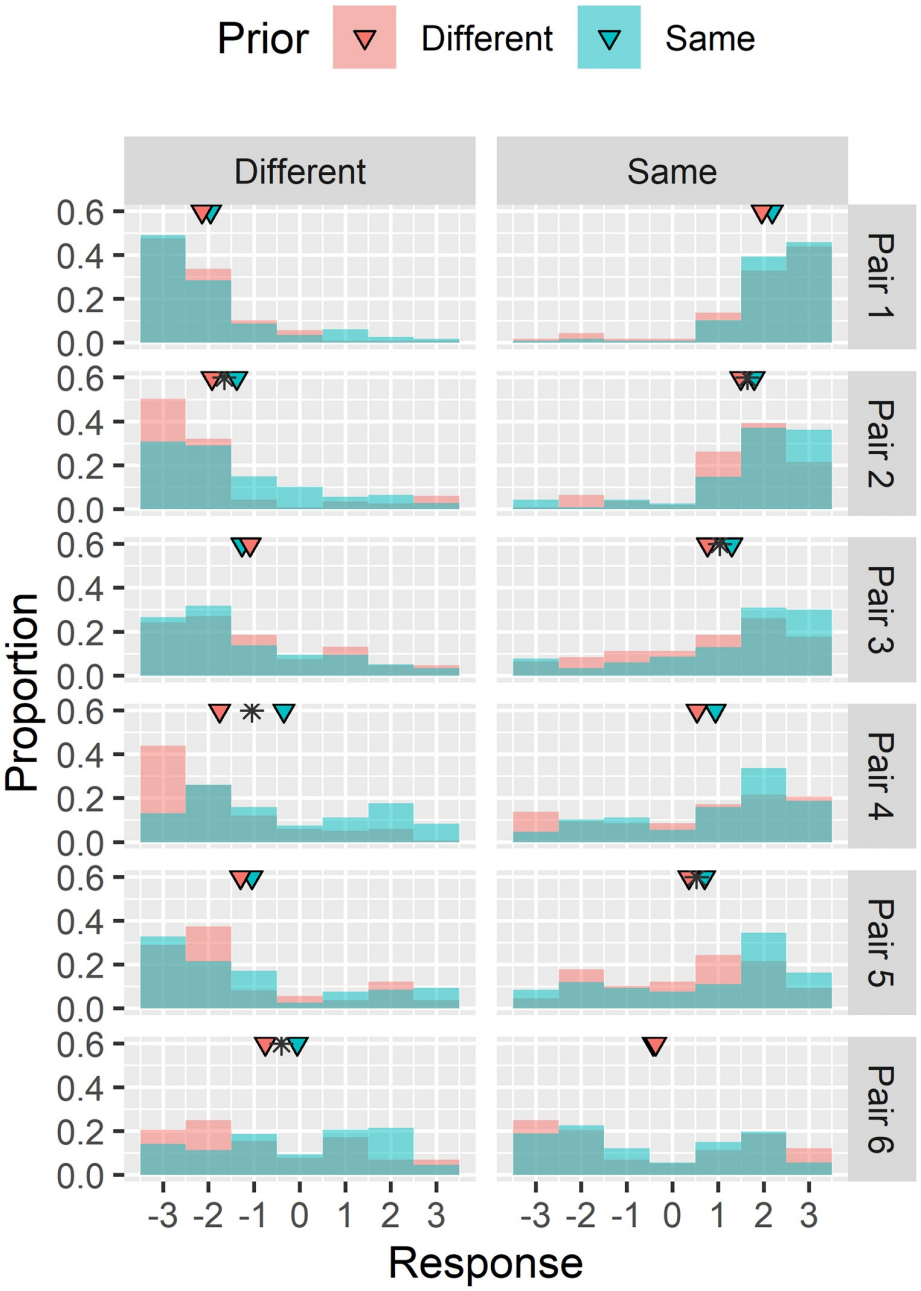
Distribution of responses for each face pair based on prior identity information content. Means of each distribution denoted with triangles. Pairs where distribution shift is significant (Wilcoxon signed-rank test) are denoted with an asterisk.

To better understand the effect of prior identity information on observed true positive and false positive rates, we analyzed the response distributions across volunteers for each face pair as a function of prior identity information: “same” or “different” (Fig 4). For each face pair, we then tested for a difference in distributions with prior identity decisions using a Wilcoxon signed-rank test. Significant differences are denoted as asterisks for each face pair in Fig 4. We found that responses were statistically different (*p* < 0.05) for 6 of the 12 individual face pairs. We quantified the magnitude of this difference by subtracting means of the two response distributions for each face pair, finding an average shift of 0.39, indicating greater confidence that the face pairs are similar when the prior identity decision was “same” versus when it was “different”. This shift was significant across all face pairs (*t*(11) = 3.34, *p* = .007). All of these findings were consistent for subjects of different age, gender, and race (see S1 Appendix).

### Signal detection theory analysis

We next examined these effects in the context of signal detection theory, which separates volunteers’ sensitivity to face similarity from any cognitive bias in making same/different judgments. Recall from our earlier discussion that, broadly, there are two ways of measuring how cognitive tasks may be impacted via SDT. The first involves changes in an observer’s sensitivity, i.e. a change in how well an observer can discriminate faces of the same individual from those of different individuals. A drop in sensitivity would be expected if adding information diverts spatial attention away from the face stimuli. Sensitivity is measured by the *d*′ metric. The second means of impacting cognitive tasks is by raising or lowering the internal criterion, i.e. the required similarity to judge a pair of faces as being of the same individual.

Table 6 shows the sensitivity (*d*′) and the criterion (cognitive bias) calculated based on average TPR and FPR estimates for each prior identity information condition. Because SDT metrics are not defined in the cases where TPR or FPR are zero or one, it was not possible to calculate SDT measures for individual subjects. We therefore examined significance by estimating 95% confidence intervals around each metric using bootstrap resampling. This analysis showed that the confidence intervals for sensitivity (*d*′) values for all three conditions overlapped, indicating that prior identity information did not influence volunteers’ ability to perceive differences in the faces as would be expected with reduced attention to the face stimulus. Instead, volunteers changed their criterion, as shown by non-overlapping confidence intervals of criterion values for the “same” and “different” conditions. This indicates that prior identity information biased the volunteers, making them more confident that two faces were of the same person in the “same” condition, and more confident that two faces were of different people in the “different” condition. These changes in response criterion were consistent for subjects of different age, gender, and race (see S1 Appendix).

**Table 6:** Sensitivity (*d*′) and criterion values as a function of prior identity decision (threshold = 0.5). 95% confidence intervals are estimated by bootstrap resampling.

Prior	n	*d*′ (95% CI)	Criterion (95% CI)
None	120	1.39 (1.24–1.55)	0.16 (0.07–0.26)
Same	223	1.24 (1.09–1.38)	0.05 (-0.03–0.13)
Different	223	1.38 (1.23–1.53)	0.27 (0.19–0.36)

So far we have considered results only for one decision threshold of *θ* = 0.5. However, we are more broadly interested in whether the effects observed in the previous sections also hold at other decision thresholds. The rating task we employed allows signal detection theory metrics to be calculated across a range of decision thresholds.

**Fig 5:**
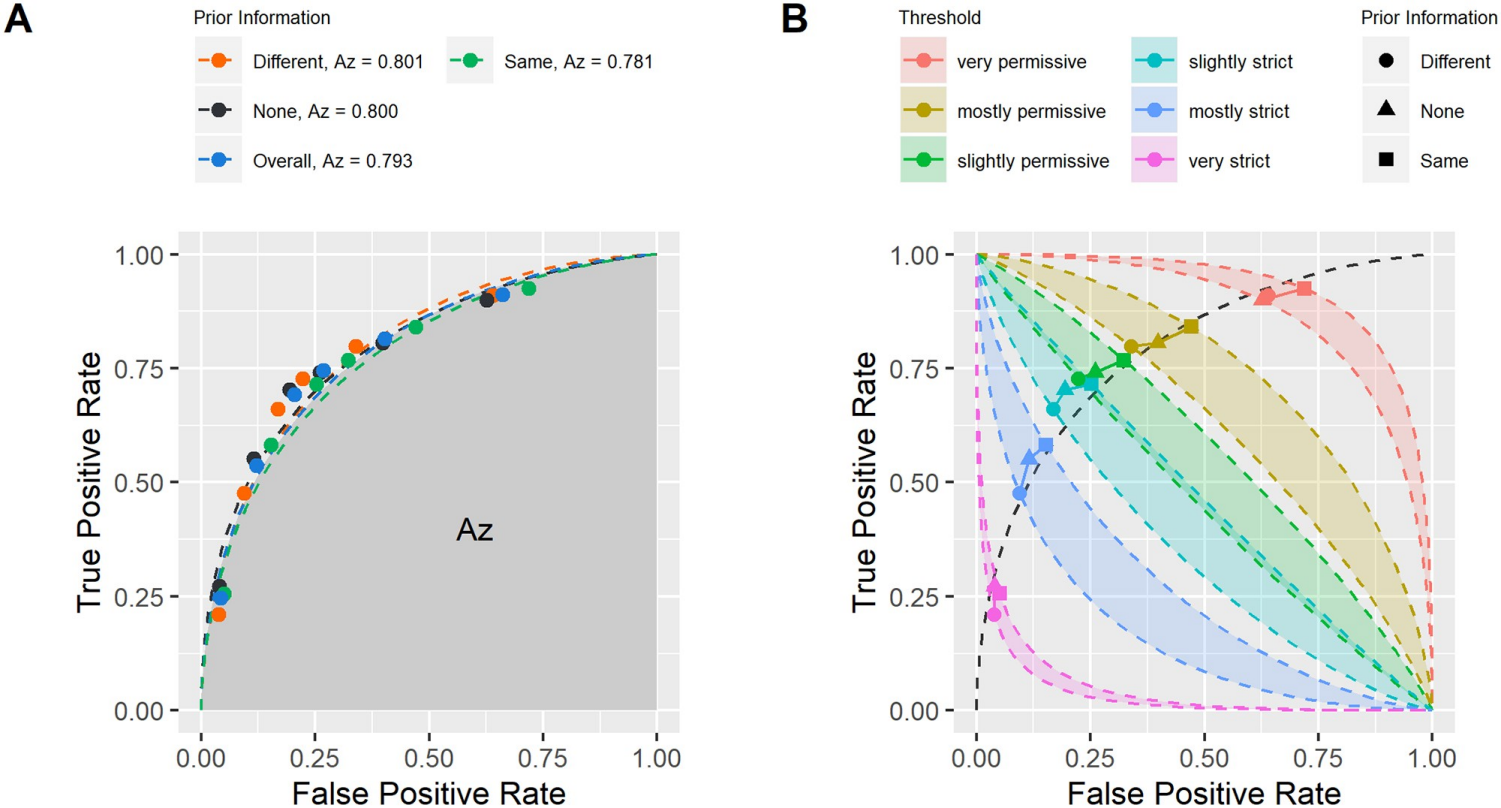
Prior identity information only modifies the receiver operating characteristic criterion. A. No change in the ROC curve with prior identity information. Dashed lines correspond to fitted ROC under gaussian approximation (linear fit in z-transformed space). Overall ROC is the ROC fitted to data collapsed across all conditions. B. Change in bias with prior identity information. Colored bands correspond to iso-bias regions spanning different prior identity information conditions for each threshold level. Note overlap in iso-bias regions for slightly permissive and slightly strict thresholds. Dashed curve is the overall ROC.

The receiver operating characteristic curves plotted in Fig 5 offer a complete visualization of the effect of prior identity information on face matching responses across thresholds, showing the measured *TPR* as a function of *FPR*. The measure of sensitivity that characterizes performance across thresholds is *Az*, which corresponds to the area under the ROC curve fitted to the data assuming a Gaussian distribution. Fig 5A plots the ROC curves as a function of prior identity information as well as for the control survey variant. All plotted points fall along the same curve with *Az* values varying by less than 0.02 indicates that prior identity information had no effect on the sensitivity across the full range of thresholds. Instead, Fig 5B shows that prior identity information biased volunteers’ criterion for making same/different judgements. Prior identity information moved responses leftward and upward along the ROC for the “same” condition and rightward and downward along the ROC for the “different” condition. Fig 5 also illustrates the magnitude of this shift. In particular, for the two middling thresholds, the shifts in response associated with prior identity information were equal to a full step on the decision scale (See Table 3). Thus, prior identity information could shift responses from “I am not sure” to “I am somewhat certain this is the same person” which could have a notable effect, especially when the match is difficult.

## Conclusion

We find that human face matching certainty decisions are systematically altered by prior identity information. When given prior identity information in the form of “same” or “different” labels, our volunteers were more confident that faces labeled “same” are similar and faces labeled “different” are different. This biased their certainty judgements of face similarity, but did not reduce their ability to discriminate faces. Interestingly, volunteers reported distrusting human identification ability more than computer identification ability. However, human and computer sources of prior identity information biased certainty responses equally. Overall, this shows that face recognition algorithms incorporated into a human process can influence human responses, likely limiting the total system performance.

Previous work on human-algorithm teaming in face recognition by Fysh and Bindemann [13] attributed increased error rates to attentional processes, postulating that algorithm match results divert attention from the face pair. Prior studies of spatial attention have shown that attentional allocation results in an increase in visual discrimination performance [26]. If match decisions diverted spatial attention away from the faces, sensitivity should have declined. However, we found no effect on sensitivity in our study, suggesting that changes in face-matching performance were not due to spatial attention. Rather, our results suggest that the biases introduced by the information carried by face-match decision labels are introduced at higher levels of decision making.

What does our study say about the likely performance of human-algorithm teams? Are they the best of both worlds, combining human ability to handle exceptions with the superior accuracy of modern face recognition algorithms? The answers likely depend on the use case. In forensic applications, trained facial examiners may carefully pore over every possible detail of available face imagery, taking months to make their decision [3]. Examiners trained to understand the inner workings of algorithms, may also learn to optimally incorporate algorithm decisions in their analytic workflow so that overall performance can be increased. Alternately, examiners may be required to make their decision independent of the algorithm and the results may be fused by a third party, reaping the demonstrated performance gains of such workflows [3]. On the other hand, in the travel environment, identity decisions must be made within a matter of seconds and human operators of face recognition systems do not have the benefit of deliberation. Further, the vast majority of algorithm face matching errors may have extenuating circumstances. In this environment, the performance of the human algorithm team may be limited by human error. For example, under time constraint, the biasing effect of a false positive result from an algorithm would reduce the likelihood that this error is caught [13]. On the other hand, given that most algorithm non-matches may have extenuating factors, humans may ultimately get in the habit of rationalizing these errors and overruling algorithm non-matches.

Much additional research is needed broadly in the area of human algorithm teaming and specifically as it applies to face matching tasks. To better estimate the total performance of human-algorithm teams, performance should be assessed in the context of actual errors made by algorithms, both in terms of frequency of error occurrence and using the specific face pairs for which errors are made. Because face recognition performance of both humans and algorithms varies with face demographics, future work should address how this influences human-algorithm teams. Doing this will require development of large, new, controlled, face pair datasets that are demographically diverse, which would allow for conclusive research on demographic effects. Further, additional research is needed regarding how and whether to present algorithm face matching decisions to humans so as to maximize total system performance. Understanding the effect of specific pieces of information, presented at specific times, during the human face matching process, should be considered before adding any information to these workflows in real world situations. Finally, face recognition is unique amongst popular biometric modalities in that humans share a similar perceptual capability. Consequently, errors made by humans and algorithms may be driven by similar underlying face features. Thus, some algorithm errors will be difficult for humans to detect. Developing face recognition algorithms that make errors that are easier for human teammates to detect could lead to better performing human-algorithm teams. With the growing adoption of face recognition technology, it is important to consider how humans and algorithms perform together and understand how these two entities interact as it may lead to the success or failure of a face matching system.

## Supporting information

S1 Appendix (PDF)
