# Supplementary material for: Human-algorithm teaming in face recognition: How algorithm outcomes cognitively bias human decision-making
Source: PLoS One. 2020 Aug 21;15(8):e0237855. doi: 10.1371/journal.pone.0237855 (PMC7444527; doi:10.1371/journal.pone.0237855)
Supplement: S1 Appendix — (PDF) [file pone.0237855.s001.pdf]

Human-algorithm teaming in face recognition: how algorithm outcomes cognitively bias human decision-making

Supplementary Material

SUPPLEMENTARY RESULTS

These supplementary results examine whether face matching task performance varied based on the demographics of our test subjects as well as based on the source of the face image pairs utilized in the study.

Face recognition accuracy and demographics

We first examined whether face matching accuracy varied based on subject race, gender, and age. Our sample size was not sufficient to measure accuracy of specific race, gender, and age combinations. Therefore, we computed accuracy independently for subjects grouped by self-reported race (White, Black or African-American, Other), gender (Male, Female), and age (≤45, >45). One subject was excluded from analysis of demographic effects due to missing information.

We performed a repeated measures ANOVA, examining the main effects of survey variant and demographic groups (between subjects) and prior identity information (within subjects) on the average accuracy with which subjects performed the face recognition task using a response threshold of 0.5 ($ACC_{0.5}$). We found significant main effects of gender ($F(1,214) = 13.2$, $p = 0.0004$) and age ($F(1,214) = 4.3$, $p = 0.04$) such that accuracy of female subjects ($ACC_{0.5} = 0.77$) was greater than accuracy of male subjects ($ACC_{0.5} = 0.71$) and accuracy of younger subjects ($ACC_{0.5} = 0.76$) was greater than accuracy of older subjects ($ACC_{0.5} = 0.72$). We found no effect of race on accuracy.

There was no significant interaction between gender or age and the source of prior identity information or whether the prior identity information was a match or no match. These data are shown graphically in Figures S1, S2, and S3 below.

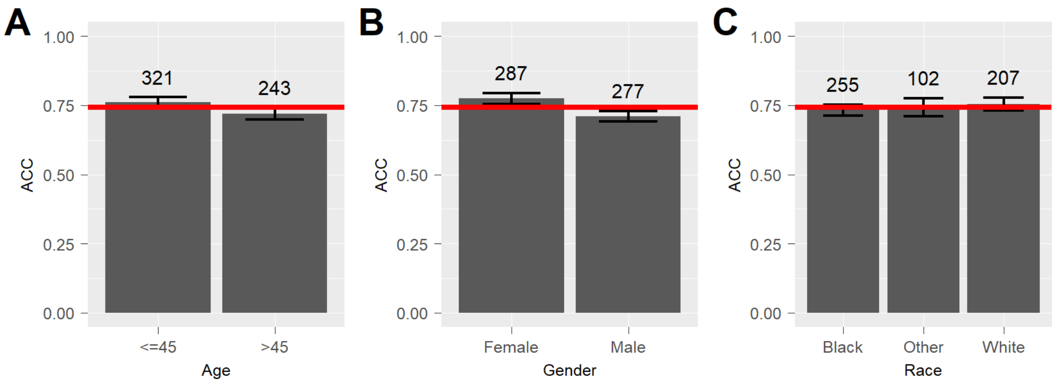

Figure S1. Accuracy ($ACC_{0.5}$) for subject subsets based on self-reported (A) age, (B) gender, and (C) race categories. Red horizontal lines show average performance across all demographics. Error bars are 95% bootstrap confidence intervals. Numbers above each bar correspond to sample size.

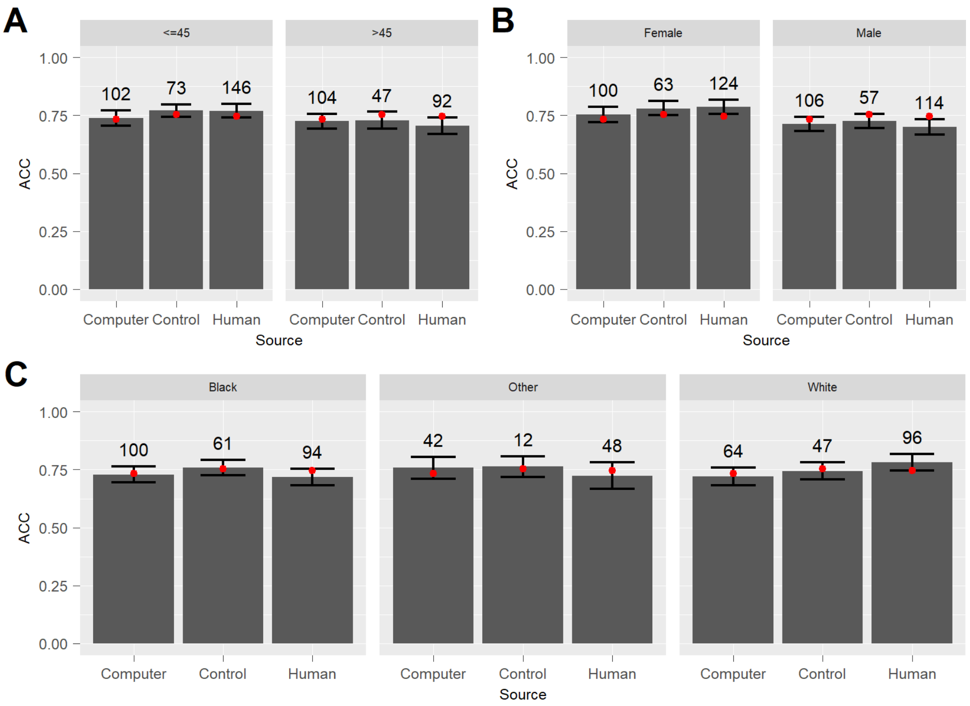

Figure S2. Accuracy ($ACC_{0.5}$) versus source (human or computer) of prior identity information for subject subsets based on self-reported (A) age, (B) gender, and (C) race categories. Red points show average performance for each demographic group across all source of prior identity information. Numbers above each bar correspond to sample size.

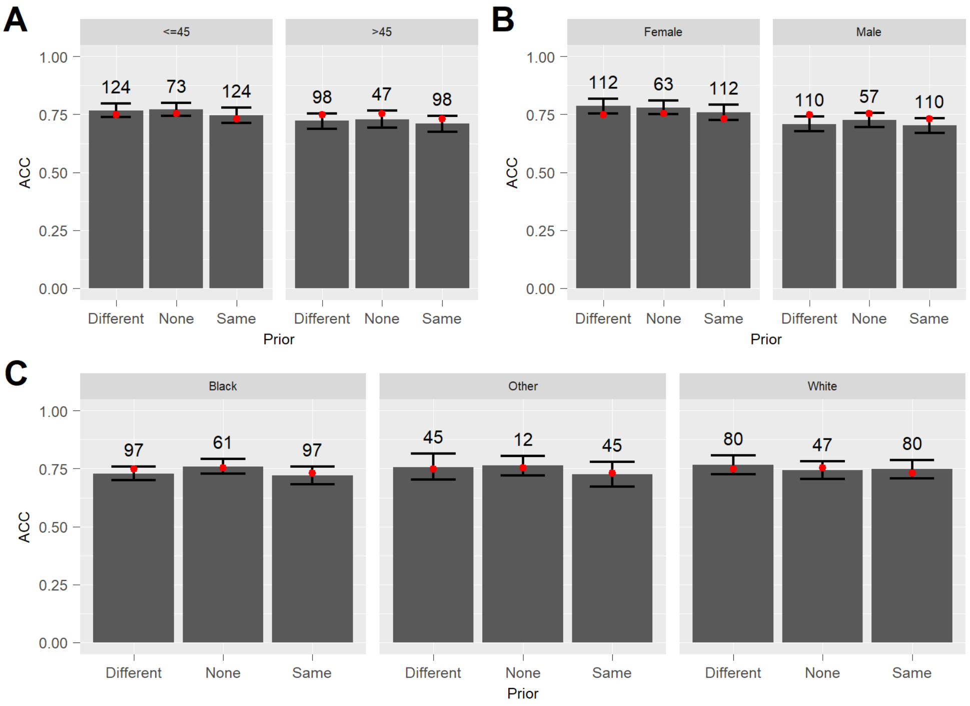

Figure S3. Accuracy ($ACC_{0.5}$) versus prior identity information provided (different, none, same) for subject subsets based on self-reported (A) age, (B) gender, and (C) race categories. Red points show average performance for each demographic group across all prior identity information conditions. Numbers above each bar correspond to sample size.

Shifts in criterion associated with prior identity information are consistent across demographic groups

We next examined whether the main effects described in our study were consistent across demographic groups. We performed repeated measures ANOVAs, examining the main effects of survey variant and demographic groups (between subjects) and prior identity information (within subjects) on the true positive rates (TPR) and the false positive rates (FPR) of the subjects using a response threshold of 0.5. In signal detection theory, these two rates determine the decision criterion such that increases in TPR and FPR are associated with a more permissive criterion and decreases in TPR and FPR are associated with a stricter criterion.

Both ANOVAs showed a significant effect of gender (TPR: $F(1,214) = 5.4$, $p = 0.021$; FPR: $F(1,214) = 5.4$, $p = 0.022$) such that TPR was higher and FPR was lower in females (females: TPR = 0.72, FPR = 0.17) relative to males (males: TPR = 0.66, FPR = 0.24) as expected from the increase in accuracy described in Figure S1. Importantly, both ANOVAs also revealed a main effect of prior identity information (TPR: $F(1,219) = 6.0$, $p = 0.015$; FPR: $F(1,219) = 15.0$, $p = 0.00014$) such that when prior identity information was "same", FPR and TPR increased to (same: TPR = 0.71, FPR = 0.25) and when it was different, FPR and TPR decreased (different: TPR = 0.66, FPR = 0.17). There were no significant main effects of age or race. No interactions between gender and prior identity information were observed in either ANOVA.

The consistency of the effects of prior identity information on subjects' decision criterion across demographic categories are summarized in Figure S4. Overall, criterion values increased (became more conservative) given "different" prior identity information and decreased (became more permissive) given "same" prior identity information consistently for all demographic groups examined. Criterion values in the absence of prior identity information ("none") were generally intermediate.

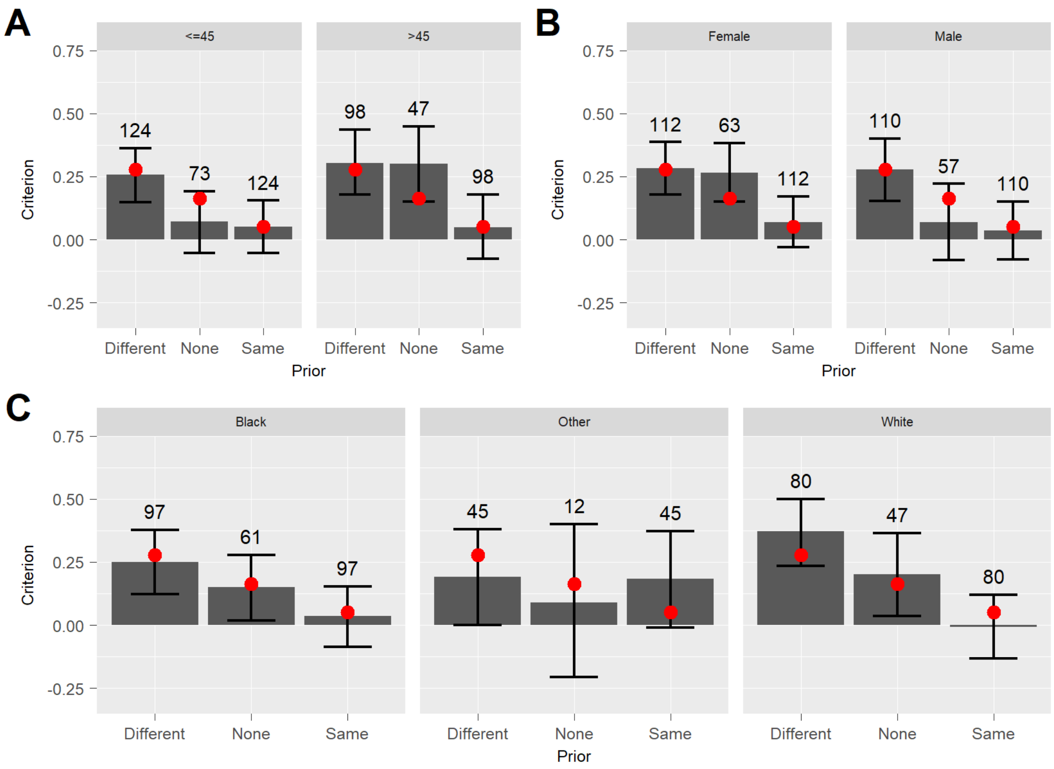

Figure S4. Decision criterion values as a function of prior identity information (different, none, same) provided for subject subsets based on self-reported (A) age, (B) gender, and (C) race categories. Red points show average criterion for each demographic group across prior identity information conditions. Numbers above each bar correspond to sample size.

Other Race Effect

A well-known phenomenon in face perception is known as the "Other Race Effect" whereby subjects perform better on face discrimination tasks when the face pairs are of the same race as the subject as compared to when the faces are of a different race (Walker and Tanaka 2003; Phillips et al. 2011). We examined whether subjects' performance on face pairs included in our face matching task was, in fact, better when the race of the subjects matched the race of the face pair.

Our face matching task included face image pairs from the Glasgow Face Matching Task (GFMT) as well as new face image pairs from the NIST Multiple Encounters Dataset (MEDS) of individuals labeled as 'Black'. The images selected from MEDS were of face pairs with no emotion and were cropped and converted to gray scale to match GFMT stimuli. Demographic information was not explicitly provided for face pairs sourced from the GFMT dataset, however, this dataset is widely understood to contain predominantly 'White' faces. We therefore grouped our subjects into those that self-identified as 'Black' and all others 'Not Black' (Subject Race) and compared the performance of these groups for 'Black Faces' from the MEDS data set and 'Not Black Faces' from the GFMT dataset (Other Race).

We performed a repeated measures ANOVA, examining the main effects of subject demographics (between subjects) and the race of the face pairs (within subjects) on the overall accuracy with which all subjects performed all face matching tasks using a response threshold of 0.5 ($ACC_{0.5}$). As previously shown in Figure S1, we found significant main effects of gender ($F(1,338) = 19.6$, $p < 0.0001$) and age ($F(1,338) = 5.9$, $p < 0.016$), but no effect of race on $ACC_{0.5}$. Additionally there was a clear main effect of face pair race ($F(1,339) = 226.8$, $p < 0.0001$) such that face pairs created from the MEDS dataset ($ACC_{0.5} = 0.86$) were easier to discriminate than face pairs from GFMT ($ACC_{0.5} = 0.68$). This is not unexpected since MEDS face pairs were selected using a computer algorithm to determine similarity whereas GFMT face pairs were selected based on human raters. Importantly, we found a significant interaction between the race of the subject and the race of the face pairs ($F(1,339) = 19.1$, $p < 0.0001$) such that black subjects performed better on black face pairs ($ACC_{0.5} = 0.88$) relative to other subjects ($ACC_{0.5} =$ 0.83) and worse on face pairs that were not black ($ACC_{0.5} = 0.66$) relative to other subjects ($ACC_{0.5} = 0.71$). This shows that the other race effect was present in our data.

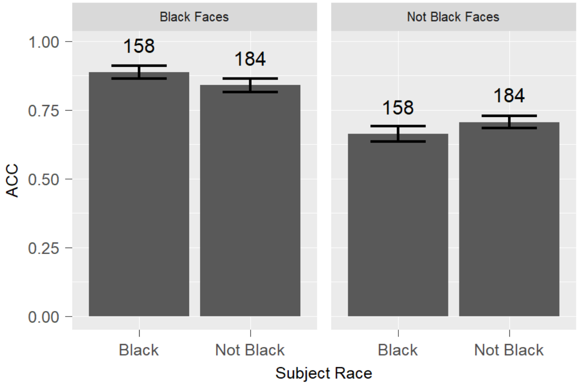

Figure S5. Improved accuracy for same-race face pairs. The left hand plot shows the average accuracy ($ACC_{0.5}$) for subjects on Black faces from the MEDS dataset. The right hand plot shows accuracy for faces from the GFMT dataset. Note relatively higher $ACC_{0.5}$ of Black subjects for Black faces from the MEDS dataset and a reversal for the GFMT dataset. Numbers above each bar correspond to sample size. Error bars are 95% bootstrap confidence intervals.

SUPPLEMENTARY DISCUSSION

We observed no broad effects on accuracy based on subject race despite the presence of the other race effect in our data. This is likely because face pairs of both races were present within each survey variant, which balanced

performance between race groups. Taken together with the lack of any effect of prior identity information and subject race on accuracy (Figure S3) and our robust observation that prior identity information affected response criterion independent of subject race (Figure S4), these findings suggest that the other race effect and the novel response bias introduced by prior identity information that we demonstrate in this work may be mediated by separate mechanisms. Indeed, the other race effect is thought to be due to perceptual learning (Walker and Tanaka 2003), which improves the neural processing of some stimuli over others, consistent with an improved sensitivity (Sagi 2011). The influence of prior identity information, on the other hand, appears restricted to the response criterion and independent of sensitivity. Thus, our study shows for our population that the effect prior identity information on performance was distinct from both long-term perceptual enhancement as well as short-term attentional mechanisms.

SUPPLEMENTARY BIBLIOGRAPHY

Phillips, P. Jonathon; Jiang, Fang; Narvekar, Abhijit; Ayyad, Julianne; O'Toole, Alice J. (2011): An other-race effect for face recognition algorithms. In *ACM Trans. Appl. Percept.* 8 (2), pp. 1–11. DOI: 10.1145/1870076.1870082.

Sagi, Dov (2011): Perceptual learning in Vision Research. In *Vision Research* 51 (13), pp. 1552–1566. DOI: 10.1016/j.visres.2010.10.019.

Walker, Pamela M.; Tanaka, James W. (2003): An encoding advantage for own-race versus other-race faces. In *Perception* 32 (9), pp. 1117–1125. DOI: 10.1068/p5098.
